# Specific Safety Profile of Bevacizumab in Asian Patients With Advanced NSCLC: A Meta-Analysis

**DOI:** 10.1097/MD.0000000000000975

**Published:** 2015-06-19

**Authors:** Zhenguang Chen, Beilong Zhong, Xueping Lun, Yingrong Lai, Amos Ela Bella, Weilin Yang, Jiabin Wu

**Affiliations:** From Department of Thoracic Surgery, the First Affiliated Hospital, Sun Yat-sen University, Guangzhou, Guangdong, China (ZC, XL); Department of Cardiothoracic Surgery of East Division, the First Affiliated Hospital, Sun Yat-sen University, Guangzhou, Guangdong, China (ZC, XL, WY, JW); Lung Cancer Research Center of Sun Yat-sen University, Guangzhou, Guangdong, China (ZC, XL, AEB, WY, JW); Department of Thoracic Surgery, the Fifth Affiliated Hospital, Sun Yat-sen University, Zhuhai, Guangdong, China (BZ); Department of Pathology, the First Affiliated Hospital, Sun Yat-sen University, Guangzhou, Guangdong, China (YL); and Department of Thoracic Surgery, Cancer Center, Sun Yat-sen University, Guangzhou, Guangdong, China (AEB).

## Abstract

Randomized studies have obtained varying findings regarding the benefits and toxicities of bevacizumab in the treatment of nonsmall cell lung cancer (NSCLC). It is unclear whether the discrepancies among trials are due to ethnic/racial differences. We therefore performed a meta-analysis of all published, randomized, controlled clinical trials involving bevacizumab in patients with NSCLC to assess its effectiveness and safety in Asian and non-Asian populations.

Results from the phase II JO19907 trial, the phase III AVAiL and ECOG 4599 trials, and the phase IV SAiL trials were used to calculate the benefits and toxicities of bevacizumab in Asian and non-Asian patients. Combined statistical estimates, including hazard ratios and odds ratios, were calculated using fixed-effects and random-effects models.

A total of 4308 patients were evaluated. Combining bevacizumab with different chemotherapy regimens resulted in similar objective response rates, overall survival, and progression-free survival in Asian and non-Asian populations. Disease control rates, however, were only reported in Asian populations. The rates of severe bleeding (relative risk [RR], 2.17; *P* = 0.02) and thromboembolism (RR, 3.65; *P* < 0.0001) were significantly higher, while the rate of severe proteinuria was significantly lower (RR, 0.43; *P* < 0.0001), in non-Asian than in Asian populations. The rates of severe hypertension (*P* = 0.71) and hemoptysis (*P* = 0.66) were similar in Asian and non-Asian populations.

Bevacizumab combined with chemotherapy for first-line NSCLC treatment showed similar benefits in Asian and non-Asian populations, but had specific safety profiles in each.

## INTRODUCTION

Primary lung cancer is a major cause of cancer-related deaths worldwide. The majority of lung-cancer patients have nonsmall cell lung cancer (NSCLC), and most patients with this aggressive and highly invasive tumor are diagnosed with advanced stage disease.^1,2^ Although platinum-based systemic chemotherapy (CTX) combinations, including cisplatin/paclitaxel, cisplatin/docetaxel, cisplatin/gemcitabine, and carboplatin/paclitaxel, have shown qualitative and quantitative benefits in patients with advanced NSCLC,^2–5^ the survival rate remains low, with only 15% of patients surviving 5 years after diagnosis.^1,6^ Many new CTX agents have been reported to improve patient survival and quality of life, but attention during the last decade has focused mainly on palliation rather than reducing mortality rates. Thus, effective treatments are required, including first-line CTX regimens, to improve patient survival.^6,7^

Vascular networks, consisting of preexisting blood vessels, have been found to form in or around tumors; these networks are regarded as active components of the tumor stroma, and mediate the transport of nutrients to tumor cells.^8^ Thus, agents that reduce tumor angiogenesis may benefit patients. Bevacizumab is a recombinant, humanized monoclonal antibody that targets all isoforms of vascular endothelial growth factor (VEGF). Clinical trials have shown that the addition of bevacizumab to CTX significantly increases the progression-free survival (PFS) and response rate of patients diagnosed with advanced NSCLC.^9,10^ Thus, bevacizumab, in combination with various CTX regimens, has been approved in the United States and Europe for the first-line treatment of patients with unresectable, locally advanced, recurrent, or metastatic nonsquamous NSCLC.^9,10^

Despite these benefits of bevacizumab in patients with advanced NSCLC, its ability to enhance overall survival (OS) remains unclear. For example, the phase III Eastern Cooperative Oncology Group (ECOG) 4599 trial showed that median OS was 2 months longer in patients treated with bevacizumab plus CTX than in patients treated with CTX alone (12.3 months vs. 10.3 months), and that the addition of bevacizumab improved the response rate (RR, 35% vs. 15%) and median PFS (6.2 months vs. 4.5 months).^11,12^ In contrast, the AVAiL (Avastin in Lung) trial found that median OS was similar in patients treated with CTX plus 7.5 and 15 mg/kg bevacizumab as in patients treated with CTX alone (13.6 months vs. 13.4 months vs. 13.1 months), with smaller improvements in median PFS (6.7 months vs. 6.5 months vs. 6.1 months) and RR (37.8% vs. 34.6% vs. 21.6%).^13,14^

These clinical trials had several limitations. First, the difference between the ethnic populations in the ECOG 4599 and AVAiL trials may be noteworthy. Patients in the AVAiL trial were from several Asian countries, including China and Thailand, whereas none of the patients in the phase III ECOG 4599 trial was from Asia. It is unclear whether discrepancies in trial results, especially median OS and PFS, are due to differences in racial/ethnic groups. More importantly, patients in these trials were not stratified by race/ethnicity, for example, into Asian and non-Asian subgroups. These limitations may have masked any racial/ethnic effects on survival outcomes, suggesting that meta-analyses should be performed to determine whether bevacizumab has advantages in certain ethnic groups.

We therefore analyzed the results of published, randomized, controlled clinical trials (RCTs) comparing the efficacy of CTX plus bevacizumab with CTX alone in patients with advanced NSCLC. Trials were analyzed if they assorted patients as Asians and non-Asians, and if they evaluated both the effectiveness and safety of bevacizumab in patients with NSCLC.

## MATERIALS AND METHODS

### Study Selection and Selection Criteria

The PubMed, Science Direct, Embase, and BMJ databases were systematically searched by computer for reports of phase II or higher RCTs published between January 2004 to April 2014 that compared bevacizumab plus CTX with CTX alone for the first-line treatment of NSCLC. In addition, abstracts published in the proceedings of the American Society of Clinical Oncology (ASCO), European Society for Medical Oncology (ESMO), and International Association for the Study of Lung Cancer (IASLC) were searched. The database search strategy used combinations of controlled descriptors from MeSH, including *bevacizumab*, *lung cancer*, *lung neoplasms*, *NSCLC*, *chemotherapy*, *therapy*, *drug therapy*, *case–control studies*, *case–base studies*, *cohort study*, and *cohort analysis*. The reference lists of all retrieved articles were also reviewed to identify additional articles missed by using these search terms.

The title and abstract of all references retrieved with these search strategies were evaluated by two researchers. Any reference with the least indication of fulfilling the inclusion criteria was listed as preselected. To minimize possible bias because of any interaction with biologic agents, we excluded trials or arms containing agents that target epidermal growth factor receptor (EGFR).

### Data Extraction

Two independent reviewers searched publications using standardized data-abstraction forms. Any discrepancy between these two reviewers was settled by an independent expert in oncology. All data were extracted directly from the text. Information collected included first author, year of publication year, targeted treatment, chemotherapy regimens, number of centers, number of patients, patient characteristics, and study design (blinded or not). Outcomes included RR, median PFS, and mean OS. The primary endpoints were PFS and OS. PFS was defined as the time from randomization to either death or disease progression, and OS as the time from randomization to death. Patients not fulfilling these criteria were censored as of last follow-up date. The incidence of toxicities was also evaluated, including the number of patients who presented with grade ≥3 adverse events (AEs), including hematological (neutropenia, thrombocytopenia, anemia, and febrile neutropenia), and nonhematological (hemoptysis, hypertension, proteinuria, venous thromboembolic events, vomiting, rash or desquamation, and epistaxis and bleeding events) AEs.^15,16^

### Statistical Analysis

Data were analyzed using the Review Manager 5.0 statistical package (Cochrane Collaboration Software).^17^ Dichotomous clinical outcomes were reported as risk ratio (RR) and survival data as hazard ratio (HR), and the corresponding 95% confidence interval (CI) and meta-analytic survival curves were calculated.^18,19^ A pooled estimate of the HR was computed with a fixed-effects model; thus, for effectiveness, an HR or RR greater than 1 favored the standard arm (CTX alone), whereas an HR or RR less than 1 favored the CTX plus bevacizumab treatment.^20^ A random-effects model was used if statistical heterogeneity was observed.^21^ The funnel plot test was performed to assess the possibility of publication bias.^22^ Funnel plot asymmetry was assessed with Egger's linear regression test using the standardized estimate of the size effect as the dependent variable and the inverse of the standard error as the independent variable. All outcomes were analyzed separately in Asian and non-Asian patients and in both combined. Results were considered statistically significant if *P* < 0.05.

## RESULTS

### First-Line Bevacizumab in Nonsmall Cell Lung Cancer

Based on our inclusion criteria, 5 eligible RCTs evaluating the effects of adding bevacizumab to CTX for the first-line treatment of NSCLC were included in our meta-analysis: the phase II JO19907 trial, the phase III AVAiL and ECOG 4599 trials, and the phase IV JO19907 and SAiL trials.^11–15,23–26^ Detailed statistical reports were available for all trials and are shown in Table 1. A total of 4308 patients were evaluated.

**TABLE 1:**
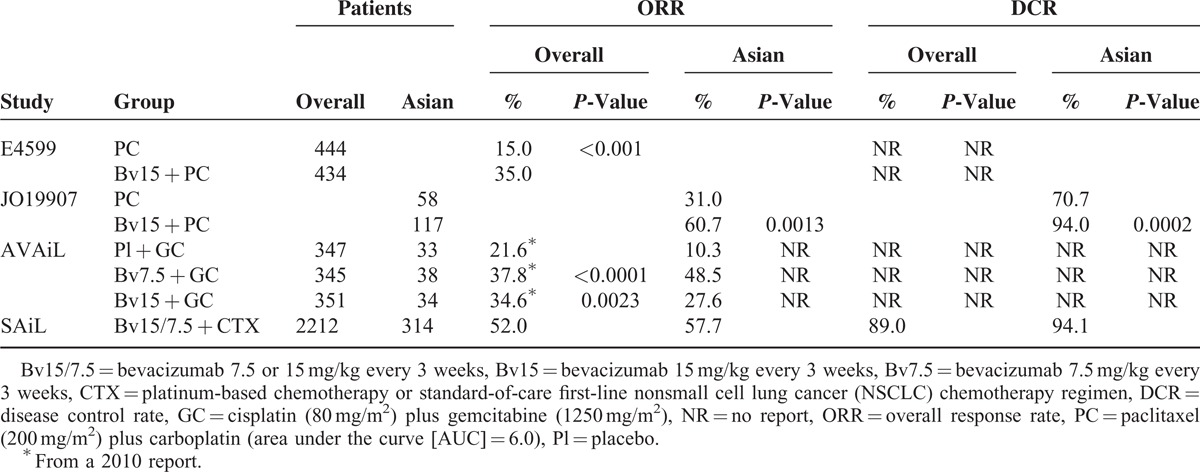
Comparisons of ORR and DCR in the Overall Study Population and in Asian Patients Enrolled in Randomized Controlled Trials of Bevacizumab Plus Chemotherapy in NSCLC

Of the 105 patients in the AVAiL trial, 91 were Asian, including 65 from Taiwan, 14 from Hong Kong (China), and 12 from Thailand; and 14 were non-Asian, including 11 from Canada and 1 each from Germany, Belgium, and Australia. There were 314 Asian patients in the SAiL trial, including 198 from China, and 175 Asian patients in the JO19907 trial. The phase III ECOG 4599 trial did not include any Asian patients.

Regarding clinical characteristics, stage IIIB NSCLC in the ECOG 4599 trial was defined as the presence of pleural effusions. All patients in the JO19907 trial had pleural effusions, pericardial effusions, and/or pleural dissemination. Both the presence of a supraventricular lymph node metastasis and malignant pleural or pericardial effusion were noted in the AVAiL trial.

In all of these trials, bevacizumab plus CTX was administered in 3-week cycles for up to 6 cycles or until evidence of disease progression or unacceptable toxic effects developed. The AVAiL and SAiL trials evaluated 2 bevacizumab doses (7.5 and 15 mg/kg), whereas the ECOG 4599 and JO19907 trials evaluated only 1 bevacizumab dose (15 mg/kg). The backbone CTX regimen was gemcitabine plus cisplatin in the AVAiL trial; paclitaxel plus carboplatin in the ECOG 4599 and JO19907 trials; and platinum-based CTX, nonplatinum doublets or single-agent CTX in the SAiL trial.

The arm designs in these trials were inconsistent. The AVAiL trial compared 7.5 mg/kg bevacizumab plus CTX, 15 mg/kg bevacizumab plus CTX, and high- or low-dose placebo plus CTX for each bevacizumab arm. However, the ECOG 4599 and JO19907 trials compared 15 mg/kg of bevacizumab plus CTX with CTX alone, that is, no placebo treatment. The primary objective of the SAiL study was to assess the safety profile of bevacizumab combined with CTX as a first-line treatment for advanced or recurrent nonsquamous NSCLC; the data were analyzed for 2 bevacizumab doses (7.5 and 15 mg/kg) plus CTX, and patients were treated with a single bevacizumab regimen until progression or unacceptable toxic effects were reported.

### Survival Profiles Associated with the Addition of Bevacizumab to Chemotherapy in Non-Asian and Asian Populations

Assessment of objective response rates (ORRs) in the ECOG 4599 trial showed a difference between patients receiving bevacizumab plus CTX and those receiving CTX alone (35.0% vs. 15.0%; Table 1). Patients in the AVAiL trial treated with bevacizumab plus CTX had a similar ORR (34.6%), whereas those in the SAiL trial had a higher ORR (52.0%). Asian patients in the JO19907 and SAiL trials treated with first-line bevacizumab plus CTX had higher ORRs (60.7% and 57.7%, respectively) than did Asian patients who received CTX alone (31.0% each). Findings from the AVAiL trial showed that ORR rates were higher in patients treated with high- or low-dose bevacizumab plus CTX (27.6% and 48.5%, respectively), than in patients treated with CTX plus placebo (10.3%).

Findings from the ECOG 4599 trial showed that median OS (12.3 months vs. 10.3 months) and median PFS (6.2 months vs. 4.5 months) were higher in patients treated with bevacizumab plus CTX than in patients treated with CTX-alone (Table 2). In contrast, the JO19907 trial showed that the addition of bevacizumab to first-line CTX significantly improved PFS (6.9 months vs. 5.9 months) and ORR (60.7% vs. 31%) compared with CTX-alone, but did not improve OS. Subsequently, the AVAiL study showed that median PFS (6.7 months vs. 6.1 months vs. 6.1 months) was similar, whereas ORR (30.4% vs. 34.1% vs. 20.1%) was greater, in patients treated with high- or low-dose bevacizumab plus CTX than in patients treated with CTX alone. Median OS was similar in the 3 arms, possibly because of treatment with an efficacious second-line regimen. The data in the SAiL trial showed a median time to progression of 7.8 months and a median OS of 14.6 months.

**TABLE 2:**
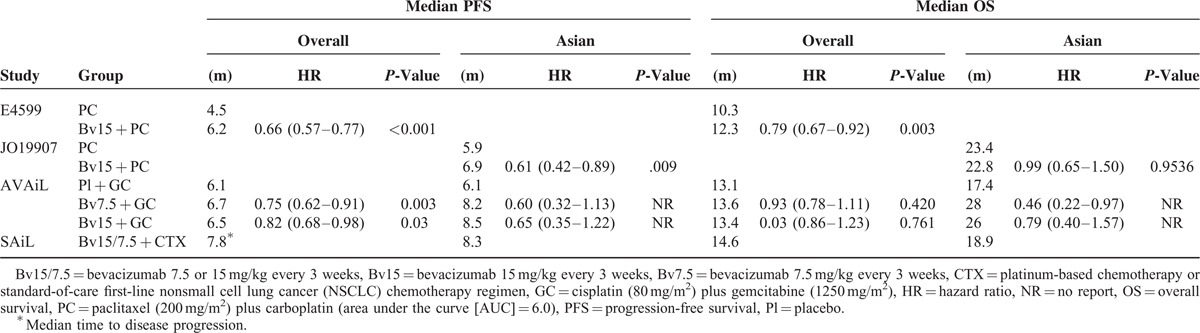
Comparisons of PFS and OS in the Overall Study Population and in Asian Patients Enrolled in Randomized Controlled Trials of Bevacizumab Plus Chemotherapy in NSCLC

Disease control rate (DCR) was only reported in the Asian population of only 1 trial, the JO19907 study. The addition of bevacizumab to first-line CTX significantly improved the DCR (94.0%) compared with the CTX-alone group (70.7%). Similarly, the DCR rate of Asian patients in the SAiL trial was 94.1%.

### Bevacizumab Safety Profiles in Asian and Non-Asian Populations

**FIGURE 1:**
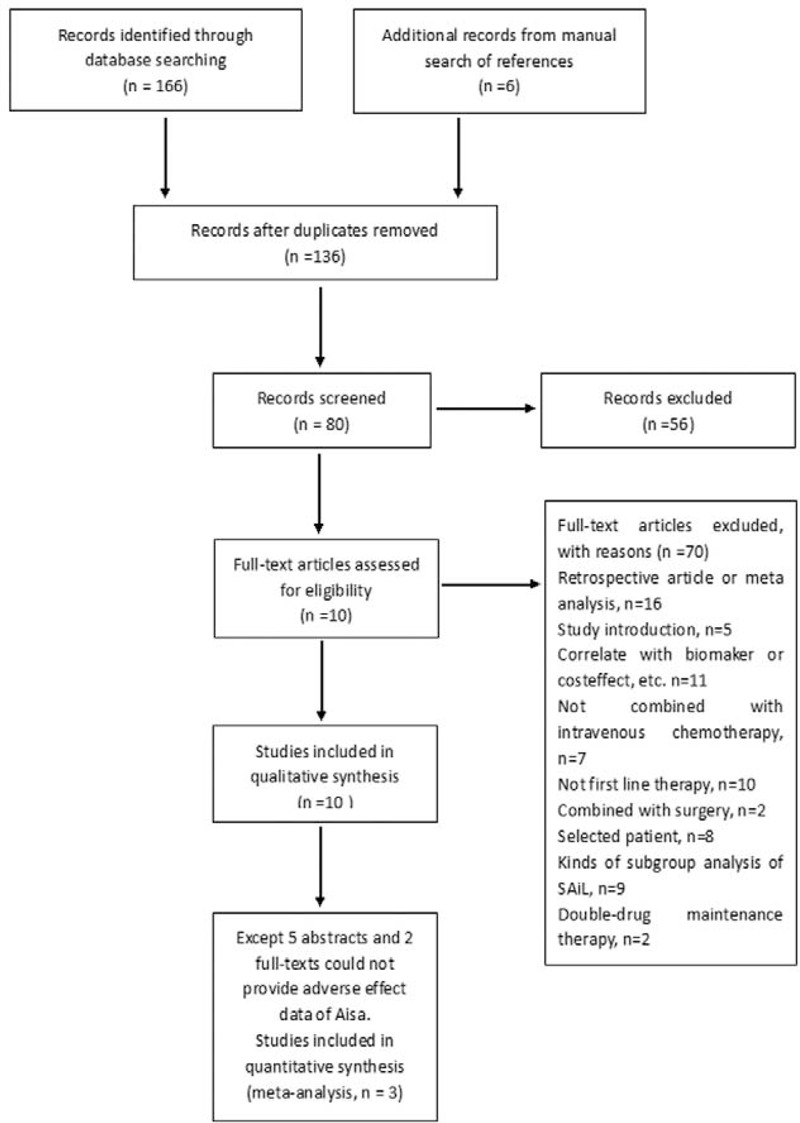
Flow chart of study selection.

Grade ≥3 bleeding events were observed in 4.4% of all patients in E4599, 4.0% of those in AVAiL, and 4.0% of patients in SAiL. In Asian populations, the rates of grade ≥3 bleeding events were 1.0% in JO19907, 3.0% in AVAiL, and 2.5% in SAiL, the latter 2 rates observed in patients treated with 15 mg/kg bevacizumab (Table 3). Meta-analysis showed that bleeding rate was significantly higher in non-Asian than in Asian patients (RR, 2.17; 95% CI, 1.14–4.15; 3356 participants; overall effect size, 2.35; *P* = 0.02; Figure 1A). In addition, grade ≥3 bleeding incidence was no higher in patients treated with high- than low-dose bevacizumab.

**TABLE 3:**
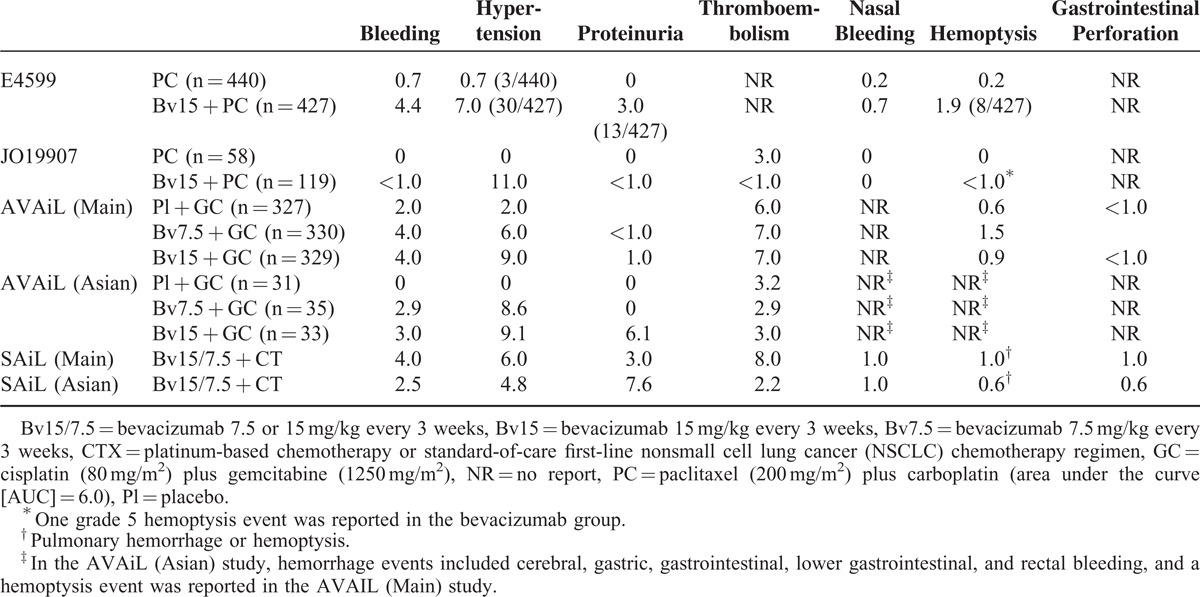
Incidence of Grade ≥3 Side Effects in NSCLC Patients Enrolled in Randomized Controlled Trials of Bevacizumab Plus Chemotherapy

The overall rates of grade ≥3 hypertension events were 7.0% in E4599, 9.0% in AVAiL, and 6.0% in SAiL after treatment with 15 mg/kg bevacizumab. In Asian patients, the rates of grade ≥3 hypertension events were 11.0% in JO19907, 9.1% in AVAiL, and 4.8% in SAiL (Table 3). The incidence of grade ≥3 hypertension was higher in Asian than in non-Asian patients, although the difference was not statistically significant (RR, 0.93; 95% CI, 0.66–1.33; 3356 participants; overall effect size, 0.37; *P* = 0.71; Figure 1B). However, the incidence of grade ≥3 hypertension higher in patients treated with 15 mg/kg than 7.5 mg/kg bevacizumab.

**FIGURE 2:**
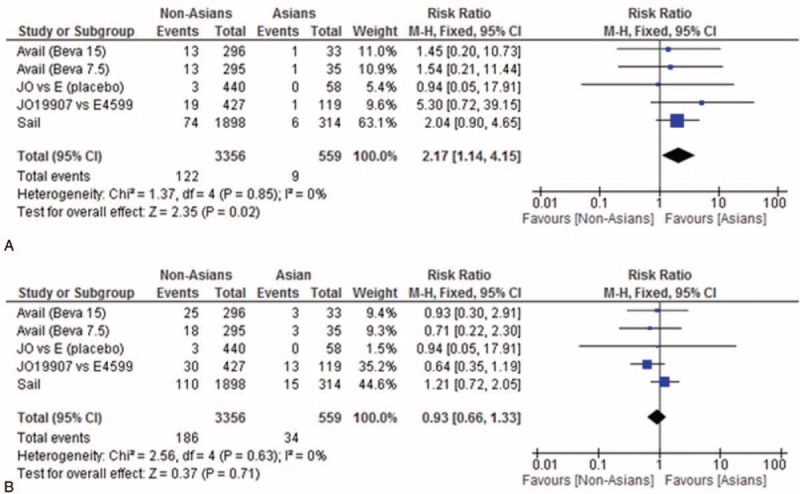
Meta-analysis of the occurrence of bleeding and hypertension in Asian and non-Asian populations. (A) The incidence of bleeding was significantly higher in non-Asian than in Asian patients (relative risk [RR], 2.17; 95% confidence interval [CI], 1.14–4.15; 3356 participants; overall effect size, 2.35; *P* = 0.02). (B) The incidence of grade ≥3 hypertension was similar in Asians and non-Asians (RR, 0.93; 95% CI, 0.66–1.33; 3356 participants; overall effect size, 0.37; *P* = 0.71).

Assessments of proteinuria profiles showed that the overall rates of grade ≥3 proteinuria were 3.0% in E4599, 1.0% in AVAiL, and 3.0% in SAiL after treatment with 15 mg/kg bevacizumab. In Asian populations, the rates of grade ≥3 proteinuria were 1.0% in JO19907, 6.1% in AVAiL, and 7.6% in SAiL (Table 3). The incidence of grade ≥3 proteinuria was significantly higher in Asian than in non-Asian patients (RR, 0.43; 95% CI, 0.28–0.66; 3356 participants; overall effect size 3.91; *P* < 0.0001; Figure 2A), but the incidence of grade ≥3 proteinuria was not associated with bevacizumab dose.

**FIGURE 3:**
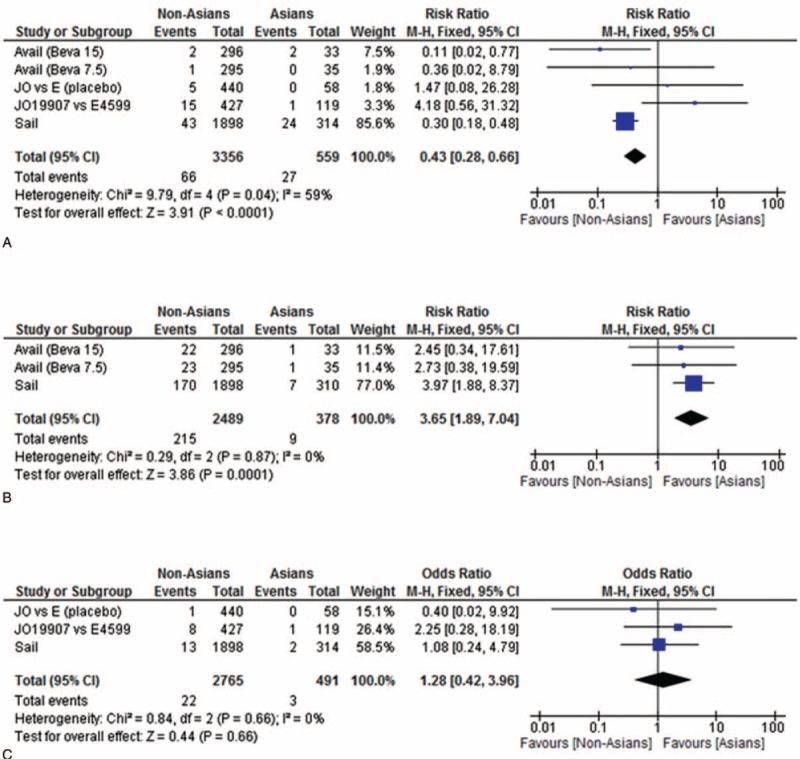
Meta-analysis of the occurrence of proteinuria, thromboembolism, and hemoptysis in Asian and non-Asian populations. (A) The incidence of grade ≥3 proteinuria was significantly higher in Asians than in non-Asians (relative risk [RR], 0.43; 95% confidence interval [CI], 0.28–0.66; 3356 participants; overall effect size, 3.91; *P* < 0.0001). (B) The incidence of grade ≥3 thromboembolism was significantly lower in Asians than in non-Asians (RR, 3.65; 95% CI, 1.89–7.04; 2489 participants; overall effect size, 3.86; *P* < 0.0001). (C) The incidence of grade ≥3 hemoptysis was similar in Asians and non-Asians (RR, 1.28; 95% CI, 0.42–3.96; 2765 participants; overall effect size, 0.44; *P* = 0.66).

Determinations of thromboembolism profiles showed that the rates of grade ≥3 thromboembolism among all patients were 0% in E4599, 6.0% in AVAiL, and 8.0% in SAiL after treatment with 15 mg/kg bevacizumab (Table 3). In Asian populations, the rates of grade ≥3 thromboembolism were 1.0% in JO19907, 3.0% in AVAiL, and 2.2% in SAiL. The incidence of grade ≥3 thromboembolism was significantly lower in Asian than in non-Asian patients (RR, 3.65; 95% CI, 1.89–7.04; 2489 participants; overall effect size, 3.86; *P* < 0.0001; Figure 2B), with no relationship between grade ≥3 thromboembolism and bevacizumab dose (Figure 3).

Rates of grade ≥3 hemoptysis in all patients were 1.9% in E4599, 0.9% in AVAiL, and 1.0% in SAiL after treatment with 15 mg/kg bevacizumab (Table 3). In Asian populations, the rates of grade ≥3 hemoptysis were 1.0% in JO19907 and 0.6% in SAiL, but these data were not reported in AVAiL. The incidence of grade ≥3 hemoptysis was similar in Asians and non-Asians (RR, 1.28; 95% CI, 0.42–3.96; 2765 participants; overall effect size, 0.44; *P* = 0.66; Figure 2C).

## DISCUSSION

Bevacizumab, a tumor-starving agent, when combined with CTX as first-line treatment for patients with advanced NSCLC, has been found to significantly improve ORR and PFS. However, bevacizumab has been associated with higher rates of severe toxicities, suggesting concerns about its safety profile. Indeed, our meta-analysis found that bevacizumab was associated with increased rates of bleeding, hypertension, proteinuria, and thrombosis.

Bleeding is an important nonhematologic event and is so prevalent that it deserves special attention. In European populations, the rates of grade ≥3 bleeding were 4% in the AVAiL trial and 4.4% in the E4599 trial, the latter including a 0.7% rate of grade ≥3 central nervous system hemorrhage. In the SAiL trial, 32%, 6%, and 4% of the patients had grades 1, 2, and grade ≥3 bleeding, respectively. More importantly, some patients in the E4599 trial died of a pulmonary or gastrointestinal hemorrhage. In the JO19907 trial, consisting of Asian patients, however, the rate of grade ≥3 bleeding was <1%, although 77% of the patients experienced grades 1 to 2 bleeding. Similarly, assessments of the Asian patients in the SAiL and AVAiL trials found that the rates of grade ≥3 bleeding were 2.5% and 2.9%, respectively, although the rates of grades 1 to 2 bleeding were 51% and 43%, respectively. These results showed a disparity in the rates of severe bleeding between Asian and European populations. However, none of the trials testing 2 doses of bevacizumab found that the incidence of grade ≥3 bleeding was dose dependent.

Another common, clinically significant adverse effect of special interest was hypertension. The rates of severe hypertension in European patients were 7% in the E4599 trial; 6% and 9% for low- and high-dose bevacizumab, respectively, in the AVAiL trial; and 6% in the SAiL trial. Among Asian patients, the rates of grade ≥3 hypertension were 11% in the JO19907 trial; 37.3% in SAiL; and 8.6% and 9.1% for low- and high-dose bevacizumab, respectively, in AVAiL. In breast cancer patients, bevacizumab-induced hypertension was reported to predict a more prolonged PFS and a higher 2-year OS rate. However, further investigations are needed to determine whether bevacizumab-induced hypertension is an indicator of better prognosis in patients with NSCLC.

Another important AE associated with bevacizumab was proteinuria. Among patients receiving 7.5 mg/kg bevacizumab plus CTX, 31% in the SAiL trial experienced grades 1 to 2 proteinuria, while 3.0% experienced grades 3 to 4 proteinuria. Similarly, 3.1% of patients in the E4599 trial and 1% of those in AVAiL experienced grades 3 to 4 proteinuria. The incidence of grades 1 to 2 proteinuria in Asian patients differed markedly among trials, being 51% in JO19907, 8.6% in AVAiL, and 39.8% in SAiL, although the rates of grades 3 to 4 proteinuria in Asians were <1% in JO19907 and 0% in AVAiL. In contrast, 7.6% of Asian patients in the SAiL trial experienced severe proteinuria. A meta-analysis of these trials found that the incidence of severe proteinuria tended to be higher in Asian than in non-Asian patients.

The rates of grades 3 to 4 venous or atrial thromboembolism among patients treated with 7.5 mg/kg bevacizumab plus CTX were 0% in the E4599 trial, 8% in SAiL, and 7% in the AVAiL, while 6% of these patients in SAiL experienced grades 1 to 2 thromboembolism. Of the patients in the AVAiL trial receiving 15 mg/kg bevacizumab plus CTX, 7% experienced grades 3 to 4 thromboembolism. Among Asian patients, 4% in JO19907, 2.9% in AVAiL, and 1.3% in SAiL experienced grades 1 to 2 thromboembolism, while <1%, 2.9%, and 2.2%, respectively, experienced grades 3 to 4 thromboembolism. These findings suggested that, compared with non-Asians, the Asian populations in these studies had higher rates of mild or moderate thromboembolism and lower rates of severe thromboembolism.

This meta-analysis had several limitations that may have complicated the interpretation of the summary statistics. For example, the pooled findings were directly driven by the strengths and weaknesses in the designs of the included studies. The included studies showed differences in response rates and inconsistent adjustments for potential confounders. In addition, like all meta-analyses, the limitations may have been due to the availability and heterogeneity of the published data, and differences in the extent to which confounding factors were controlled. For example, this analysis was performed using published study results rather than data from individual patients. The control arms in the two studies with more than one bevacizumab-containing arm had to be duplicated for independent statistical comparisons. Moreover, the definition of PFS was not precisely specified in all studies.

A clear understanding of the magnitude of the benefits and toxicities of bevacizumab in subgroups of patients with NSCLC is of paramount importance. Correct estimates of treatment-related toxicities and the efficacy of bevacizumab are fundamental to provide appropriate guidance and to conduct ongoing trials.

## CONCLUSION

Bevacizumab combined with chemotherapy for first-line NSCLC treatment showed similar benefits in Asian and non-Asian populations, but had specific safety profiles in each. Compared with non-Asian population, there were higher rates of severe bleeding and thromboembolism and lower rate of severe proteinuria in Asian population.
